# Spray-dried blood plasma in broiler nutrition improves performance, intestinal health, and carcass traits

**DOI:** 10.1016/j.psj.2026.106556

**Published:** 2026-01-30

**Authors:** Luzia Trajano da Silva, Adiel Vieira de Lima, Danilo Vargas Gonçalves Vieira, Danilo Teixeira Cavalcante, Matheus Ramalho de Lima, Apolônio Gomes Ribeiro, Carlos Henrique do Nascimento, Paloma Eduarda Lopes de Souza, Aline Beatriz Rodrigues, Ricardo Romão Guerra, Leonardo Augusto Fonseca Pascoal, Lucas Rannier Ribeiro Antonino Carvalho, Fernando Guilherme Perazzo Costa

**Affiliations:** a Federal University of Paraíba, Department of Animal Science, Areia, Paraíba, Brazil; b Federal University of Northern Tocantins, Department of Animal Science, Araguaína, Tocantins, Brazil; c Federal University of Agreste of Pernambuco, Department of Animal Science, Garanhuns, Pernambuco, Brazil; d Federal Rural University of the Semi-Arid Region, Mossoró, Rio Grande do Norte, Brazil; e Department of Physiology and Pharmacology, Karolinska Institutet, Biomedicum 5B, Solnavägen 9, S-171 77, Stockholm, Sweden

**Keywords:** Immunoglobulins, Intestinal histology, Gut microbiota, Nutrition, Performance

## Abstract

This study evaluated the inclusion of spray-dried porcine plasma in the diets of broiler chickens derived from breeder hens of different ages (36 and 56 wk), reared under reused litter and high stocking density conditions (12 broilers/m²). A total of 6,000 one-d-old Cobb®500 broilers were randomly assigned to a completely randomized design in a 2 × 4 factorial arrangement: two breeder ages and four plasma inclusion levels (0%, 0.25%, 0.5%, and 1.0%). Evaluations were conducted across five growth phases: 1–7, 8–14, 15–21, 22–33, and 34–44 d, assessing performance, intestinal morphology, microbiological profile, and economic viability. Breeder age significantly influenced final body weight, weight gain, and feed conversion ratio up to 21 d, as well as villus height up to 44 d and crypt depth at d 7, 21, and 44 d. Effects were also observed on breast and eviscerated carcass weight and yield. Plasma inclusion positively impacted performance up to 14 d, improving weight gain, feed intake, and all histological parameters except goblet cell count, which was affected only up to 21 d. Additionally, plasma influenced breast and carcass traits. A significant interaction between breeder age and plasma inclusion was found for final body weight, weight gain up to 14 d, feed intake across all phases, and mortality rate. Microbiological analysis revealed the presence of Escherichia coli and Salmonella spp. in various biological samples throughout the experiment, with higher incidence at d 21 and 44. Dietary inclusion of porcine plasma improved intestinal morphology and zootechnical performance, with optimal supplementation up to 7 d for broilers from older breeders and up to 21 d for those from younger breeders.

## Introduction

The rapid expansion of poultry production in recent years, driven by the growing global demand for chicken meat, has placed increasing pressure on production systems. According to the Food Outlook, 2025 report, global poultry meat production is projected to reach 152 million tons, reflecting a 1.72% increase compared to the previous year. This growth is attributed to strong international demand and the competitive advantage of chicken meat over other animal protein sources (FAO, 2025).

Consequently, a significant rise in broiler stocking density has been observed, leading to management-related challenges and welfare concerns (Shynkaruk et al., 2023). One widely adopted strategy to mitigate waste generation is the reuse of poultry litter, a common practice in several countries, including Brazil (Garces-Gudiño et al., 2018). However, litter reuse requires strict sanitary protocols, including thorough cleaning and disinfection of facilities, and remains a limiting factor for international trade due to the potential risk of pathogen dissemination.

Following the European Union’s ban on antibiotic growth promoters (Effective 2006 under Regulation (EC) No 1831/2003) (European Commission, 2003, 2005), there is an urgent need to identify effective alternatives.

There is an urgent need to identify effective alternatives for bacterial load control in poultry systems. Among these alternatives, spray-dried plasma (SDP) has gained attention for its ability to reduce bacterial proliferation under high sanitary challenge conditions (Blue et al., 2023).

Dietary inclusion of SDP has shown beneficial effects under environmental stress (Koelkebeck et al., 2014) and during naturally occurring necrotic enteritis (Campbell et al., 2006). High stocking density itself is a known risk factor for poorer welfare and performance (Shynkaruk et al., 2023).

Therefore, the objective of this study was to evaluate the effects of dietary inclusion of spray-dried porcine plasma in broiler chickens derived from breeder flocks of different ages (36 and 56 wk), reared on reused litter under high stocking density conditions.

## Materials and methods

All experimental procedures involving animals were conducted in accordance with ethical standards and approved by the Animal Use Ethics Committee of the Federal University of Paraíba (UFPB), Campus II, Areia–PB, Brazil. The study was registered under protocol number CEUA/UFPB n°1426110924, ensuring compliance with national and institutional guidelines for the care and use of animals in research.

### Housing and experimental scheme

The experiment was conducted at the Poultry Research Facilities of the Center for Agricultural Sciences, Campus II, Federal University of Paraíba, located in Areia, Paraíba, Brazil. A total of 6,000 male Cobb®500 broiler chicks were used, originating from breeder flocks of two distinct ages: 1,500 broilers from 36-wk-old breeders and 4,500 broilers from 56-wk-old breeders.

Broilers were housed in a poultry barn containing 80 experimental unit (EUs) measuring 1.50 *m* × 1.50 m (2.25 m²), using recycled litter and a stocking density of 12 broilers/m² or 38.4 kg/m² (based on a final body weight of 3.2 kg at 42 d), totaling 27 broilers per EUs. Of the total 6,000 broilers, 2,160 were allocated to the 80 in EUs, while the remaining 3,840 broilers were reared loose within the barn to simulate commercial conditions and increase sanitary challenge exposure.

Throughout the experimental period, broilers had ad libitum access to feed and water. A continuous lighting program (24 h of light) was adopted. All broilers were vaccinated against infectious bursal disease (Gumboro®) and Newcastle disease at 7 d of age. The evaluated phases were: 1–7, 8–14, 15–21, 22–33, and 34–44 d.

The experiment followed a completely randomized design analyzed by phase (1–7, 8–14, 15–21, 22–33, and 34–44 d) as a 2 × 4 factorial: breeder age (36 vs 56 wk) and spray-dried plasma (SDP) feeding program (T1 = 0%; T2 = 1.0% to 7 d; T3 = 1.0% to 7 d then 0.5% to 14 d; T4 = 1.0% to 7 d, 0.5% to 14 d, and 0.25% to 21 d). Each treatment had 10 replicates with 27 broilers/EU; thus, the pen was the experimental unit for performance and carcass traits.

Treatments T_1_ to T_4_ included broilers from 36-wk-old breeders. Broilers in T_1_ received no plasma supplementation during any phase. T_2_ – broilers received 1.0% plasma until 7 d. T_3_ – broilers received 1.0% plasma until 7 d and 0.5% until 14 d. T4 – broilers received 1.0% plasma until 7 d, 0.5% until 14 d, and 0.25% until 21 d.

Treatments T_5_ to T_8_ included broilers from 56-wk-old breeders. T_5_ – broilers received no plasma supplementation. T_6_ – broilers received 1.0% plasma until 7 d. T_7_ – broilers received 1.0% plasma until 7 d and 0.5% until 14 d. T_8_ – broilers received 1.0% plasma until 7 d, 0.5% until 14 d, and 0.25% until 21 d. From d 22 to d 44, no plasma was included in the diets for any treatment group.

Within each phase, data were analyzed by two-way ANOVA using the model

Y_ijk_ = μ+ Ba_i_ + SDP_j_ + (BA × SDP)_ij_ + ε_ijk_​, where BA is breeder age (*i* = 1,2), SDP is feeding program (*j* = 1, 2, 3 and 4), and ε is the residual error. Residuals were checked for normality and homoscedasticity; transformations were applied as needed. When factor effects were significant (α=0.05), means were separated by Tukey’s HSD.

In addition to ANOVA, we tested a priori single-df contrasts to address specific nutrition questions: C1: T1 *vs* mean (T2–T4) — *any SDP vs none*; C2: T1 *vs* T2 — *minimal program (1.0% to 7 d) vs none*; C3: T2 *vs* T3 — *extending SDP to 14 d*; C4: T3 vs T4 — *extending SDP to 21 d*. Contrasts were evaluated using the model mean square error at α = 0.05. (Because the set C1–C4 exceeds the three available df for the SDP factor, these are preplanned, non-orthogonal contrasts).

Presence/absence of Escherichia coli and Salmonella spp. in litter, ceca, and cloacal swabs was summarized descriptively (no inferential statistics), as samples were pooled at the treatment level.

For intestinal histomorphometry, seven birds per treatment and age (1, 7, 14, 21, and 44 d) were sampled; the individual bird was considered the experimental unit.

At 7, 14, 21, and 44 d, performance parameters, histology, microbiology, carcass characteristics, and economic evaluation were measured.

Treatments were randomly assigned to experimental units following a completely randomized design. Diets (Table 1, Table 2) were formulated according to the nutritional recommendations of Rostagno et al. (2011).

**Table 1:** Proximate and chemical composition of experimental diets for broiler chickens from 1 to 21 d.

Ingredients (g/kg)	T1/T5	T2/T3/T4 T6/T7/T8	T1/T2 T5/T6	T3/T4 T7/T8	T1/T2/T3 T5/T6/T7	T4/T8
Age (d)	01–07	01–07	08–14	08–14	15–21	15–21
Corn	556.53	571.27	628.32	635.70	628.32	640.11
Soybean meal	380.69	362.50	299.22	290.13	299.22	287.40
Meat and bone meal	—	—	40.00	40.00	40.00	40.00
Spray-dried plasma	—	10.00	—	5.00	—	2.50
Soybean oil	20.00	15.08	11.71	9.25	11.71	9.00
Dicalcium phosphate	19.07	18.54	2.76	2.49	2.76	2.70
Limestone	8.83	9.23	5.42	5.63	5.42	5.61
Common salt	5.07	4.52	4.12	3.84	4.12	3.98
DL-Methionine	3.56	3.37	2.92	2.82	2.92	2.93
L-Lysine HCl	2.88	2.57	2.70	2.55	2.70	2.84
L-Threonine	1.06	0.82	0.78	0.67	0.78	0.82
L-Valine	0.76	0.54	0.49	0.38	0.49	0.55
Choline chloride	0.70	0.70	0.70	0.70	0.70	0.70
Mineral premix†	0.50	0.50	0.50	0.50	0.50	0.50
Vitamin premix*^‡^*	0.25	0.25	0.25	0.25	0.25	0.25
Antioxidant	0.10	0.10	0.10	0.10	0.10	0.10
Total (%)	1000.0	1000.0	1000.0	1000.0	1000.0	1000.0

Nutrient (g/kg)	T1/T5	T2/T3/T4 T6/T7/T8	T1/T2 T5/T6	T3/T4 T7/T8	T1/T2/T3 T5/T6/T7	T4/T8
Crude protein	222.0	222.0	208.0	208.0	208.0	208.0
Metabolizable energy (MJ/kg)	12.33	12.33	12.55	12.55	12.55	12.55
Available phosphorus	4.70	4.70	3.91	3.91	3.91	3.91
Calcium	9.20	9.20	8.19	8.19	8.19	8.19
Sodium	2.20	2.20	2.10	2.10	2.10	2.10
Chloride	4.23	3.93	3.85	3.71	3.85	3.83
Potassium	8.58	8.32	7.57	7.44	7.57	7.39
Digestible lysine	13.10	13.10	11.74	11.74	11.74	11.74
Digestible Met+Cys	9.44	9.44	8.46	8.46	8.46	8.46
Digestible threonine	8.52	8.52	7.63	7.63	7.63	7.63
Digestible valine	10.09	10.09	9.04	9.04	9.04	9.04
Digestible isoleucine	8.64	8.62	7.62	7.61	7.62	7.49
Digestible tryptophan	2.48	2.52	2.13	2.15	2.13	2.10
Digestible arginine	13.96	13.90	12.77	12.74	12.77	12.55
Digestible leucine	17.15	17.48	16.06	16.23	16.06	15.98
Digestible Phe+Tyr	17.42	17.63	15.56	15.67	15.56	15.39
Digestible histidine	5.43	5.53	4.91	4.96	4.91	4.87

† Premix mineral — teor por kg de produto Ferro (Fe) – 100 g; Cobalto (Co) – 2.0 g; Cobre (Cu) – 20 g; Manganês (Mn) – 160 g; Zinco (Zn) – 100 g; Iodo (I) – 2.0 g; Veículo/inerte q.s.p. 1,000 g. Contribuição na ração (inclusão: 1 kg/*t* = 1 g/kg): Fe 100 mg/kg; Co 2 mg/kg; Cu 20 mg/kg; Mn 160 mg/kg; Zn 100 mg/kg; I 2 mg/kg. Vit. A – 10,000,000 UI; Vit. D₃ – 2,000,000 UI; Vit. E – 30,000 UI; Vit. B₁ – 2.0 g; Vit. B₂ – 6.0 g; Vit. B₆ – 4.0 g; Vit. B₁₂ – 0.015 g; Ác. pantotênico – 12.0 g; Biotina – 0.1 g; Vit. K₃ – 3.0 g; Ác. fólico – 1.0 g; Niacina – 50.0 g; Selênio – 250 mg; Veículo/inerte q.s.p. 1,000 g. Contribuição na ração (inclusão: 1 kg/*t* = 1 g/kg): Vit. A 10,000 UI/kg; D₃ 2,000 UI/kg; E 30 UI/kg; B₁ 2 mg/kg; B₂ 6 mg/kg; B₆ 4 mg/kg; B₁₂ 0.015 mg/kg; Pantotênico 12 mg/kg; Biotina 0.10 mg/kg; K₃ 3 mg/kg; Fólico 1 mg/kg; Niacina 50 mg/kg; Se 0.25 mg/kg.

**Table 2:** Proximate and chemical composition of experimental diets for broiler chickens.

Ingredients (g/kg)	22–33 d	34–44 d
	All treatments	All treatments
Corn	643.19	660.66
Soybean meal	269.42	263.66
Meat and bone meal	40.00	10.00
Spray-dried plasma	—	—
Soybean oil	31.37	39.94
Dicalcium phosphate	10.42	7.64
Limestone	4.84	6.58
Common salt	3.87	4.27
DL-Methionine	2.62	2.41
L-Lysine HCl	2.42	2.40
L-Threonine	0.59	0.53
L-Valine	0.40	0.36
Choline chloride	0.70	0.70
Mineral premix†	0.50	0.50
Vitamin premix*^‡^*	0.25	0.25
Antioxidant	0.10	0.10
Total (%)	100.0	100.0
Nutrient (g/kg)	22–33 d	34–44 d
Crude protein	195.0	180.0
Metabolizable energy (MJ/kg)	13.18	13.48
Available phosphorus	3.42	2.98
Calcium	7.32	6.38
Sodium	2.00	1.95
Chloride	3.65	3.71
Potassium	7.06	6.81
Digestible lysine	10.78	10.10
Digestible Met+Cys	7.87	7.37
Digestible threonine	7.01	6.56
Digestible valine	8.41	7.88
Digestible isoleucine	7.08	6.74
Digestible tryptophan	1.96	1.88
Digestible arginine	11.87	10.89
Digestible leucine	15.24	14.57
Digestible Phe+Tyr	14.54	3.86
Digestible histidine	4.61	4.40

† Premix mineral — teor por kg de produto Ferro (Fe) – 100 g; Cobalto (Co) – 2.0 g; Cobre (Cu) – 20 g; Manganês (Mn) – 160 g; Zinco (Zn) – 100 g; Iodo (I) – 2.0 g; Veículo/inerte q.s.p. 1,000 g. Contribuição na ração (inclusão: 1 kg/*t* = 1 g/kg): Fe 100 mg/kg; Co 2 mg/kg; Cu 20 mg/kg; Mn 160 mg/kg; Zn 100 mg/kg; I 2 mg/kg. Vit. A – 10,000,000 UI; Vit. D₃ – 2,000,000 UI; Vit. E – 30,000 UI; Vit. B₁ – 2.0 g; Vit. B₂ – 6.0 g; Vit. B₆ – 4.0 g; Vit. B₁₂ – 0.015 g; Ác. pantotênico – 12.0 g; Biotina – 0.1 g; Vit. K₃ – 3.0 g; Ác. fólico – 1.0 g; Niacina – 50.0 g; Selênio – 250 mg; Veículo/inerte q.s.p. 1,000 g. Contribuição na ração (inclusão: 1 kg/*t* = 1 g/kg): Vit. A 10,000 UI/kg; D₃ 2,000 UI/kg; E 30 UI/kg; B₁ 2 mg/kg; B₂ 6 mg/kg; B₆ 4 mg/kg; B₁₂ 0.015 mg/kg; Pantotênico 12 mg/kg; Biotina 0.10 mg/kg; K₃ 3 mg/kg; Fólico 1 mg/kg; Niacina 50 mg/kg; Se 0.25 mg/kg.

### Growth performance assessment

Performance parameters were assessed by measuring feed intake, body weight gain, feed conversion ratio, and final body weight. Mortality was recorded daily and calculated as the ratio between the number of dead broilers during the experimental period and the initial number of broilers per replicate, multiplied by 100.

### Histological analyses

For histological analyses, at the end of each phase (7, 14, 21, and 44 d), biological samples of the small intestine (duodenum, jejunum, and ileum) were collected from seven broilers per treatment to evaluate intestinal parameters (villus height, crypt depth, villus-to-crypt ratio, and number of goblet cells).

Samples were fixed in Metacarn for 12 h embedded in paraffin following standard histological procedures. Image digitization and morphometric measurements were performed using the Motic Image software and a Motic digital camera attached to an Olympus BX-40 microscope. To assess villus height and crypt depth, four photomicrographs per sample were captured using a 4 × objective lens, with one villus and its corresponding crypt measured in each image. Goblet cell quantification was performed on transverse sections of intestinal samples from seven broilers per treatment, allowing visualization of intestinal villi and per lumen. Multiple photomicrographs were captured using a 20 × objective lens, and at least two images per broiler were randomly selected. The intestinal epithelium was measured linearly until reaching 2000 µm.

Within these measured epithelial areas, goblet cells were counted using periodic acid-Schiff (PAS) staining, which stains goblet cells magenta. Based on these results, the number of goblet cells per 1000 µm of intestinal epithelium was determined for each treatment.

### Microbiological analysis

Microbiological sampling was performed at five distinct time points: 0 (1 d), 1 (7 d), 2 (14 d), 3 (21 d), and 4 (44 d). At each time point, samples were collected from poultry litter, cecal contents, and cloacal swabs from broiler chickens of corresponding ages.

Cecal contents were obtained from three broilers per treatment group following euthanasia by cervical dislocation, in accordance with Resolution No. 1000 of the Brazilian Federal Council of Veterinary Medicine (CFMV). Samples were pooled per treatment and collected under laminar flow using sterile forceps and scissors.

Cloacal swabs were collected using sterile swabs, with one swab used for every two broilers. A total of five swabs were collected from ten broilers per treatment, pooled, stored in sterile containers, and transported under refrigeration.

For Escherichia coli detection, a pre-enrichment protocol was employed. Litter samples (25 g) were homogenized in 225 mL of 1.0% buffered peptone water, while cecal contents and cloacal swabs were incubated in 10 mL of 1.0% buffered peptone water at 37°C for 24 h. Subsequently, samples were streaked onto Eosin Methylene Blue (EMB) agar plates. Two typical colonies were selected and subjected to biochemical characterization using Triple Sugar Iron (TSI) agar, Simmons Citrate, Methyl Red (MR), Voges-Proskauer (VP), Indole, motility, and hydrogen sulfide production in SIM medium, following Carter (1988).

For Salmonella spp. isolation, 1 mL of the pre-enriched samples was transferred to 10 mL of Tetrathionate (TT) broth and 0.1 mL to Rappaport-Vassiliadis (RV) broth, both incubated under the same conditions. Cultures were then streaked onto Xylose Lysine Deoxycholate (XLD) agar and Brilliant Green Agar (BGA).

Typical colonies were further tested using Lysine Iron Agar (LIA), TSI agar, and Urea broth, incubated at 37°C for 24 hours to confirm genus identity, as described by Carter (1988).

### Carcass yield analysis

At 44 d, three broilers per experimental unit were slaughtered based on the average body weight of each replicate. Carcass yield was calculated using the weight of the whole carcass, including feet, head, and neck, to reflect commercial processing standards.

Breast yield was determined using the weight of the bone-in breast. Abdominal fat yield (%) was calculated by weighing the total fat content present in the abdominal cavity and gizzard, representing the overall fat deposition in the visceral region.

All components were weighed using a precision digital scale, and results were expressed as percentages relative to live body weight. These metrics were used to evaluate the influence of dietary treatments on carcass composition, muscle development, and fat accumulation.

### Data Analysis

All results were subjected to analysis of variance (ANOVA) at a significant level of α = 0.05. Microbiological data were evaluated using descriptive statistics to determine the presence or absence of *Escherichia coli* and *Salmonella* spp. Subsequently, treatment comparisons were performed using non-orthogonal contrasts and Tukey’s test. Statistical analyses were conducted using the SAEG software (System for Statistical and Genetic Analyses), developed by the Federal University of Viçosa (UFV, 2007).

## Results and discussion

No interaction effect was observed between breeder age and dietary plasma inclusion on broiler performance (Table 3). However, dietary inclusion of blood plasma significantly affected (*P* < 0.05) all performance parameters evaluated in broiler chickens. Breeder age influenced broilers performance up to 21 d, particularly final body weight, weight gain, and feed conversion ratio. Broilers from older breeders showed superior results compared to those from younger breeders (Table 3).

**Table 3:** Final body weight (FBW, g), feed intake (FI, g), weight gain (WG, g), conversion ratio (FCR), and mortality (M, %) of broiler chickens from 1 to 44 d fed diets containing blood plasma.

Parameters	D	Breeder age			†Inclusion of plasma in the diet					^¶^Contrasts				CV
		Wk		*^‡^P*										
		36	56		T1	T2	T3	T4	*^§^P*	C1	C2	C3	C4	
FBW	01/07	232	245	**	235	239	239	239	**	**	**	ns	ns	1.0
	01/14	580	610	**	578	598	599	605	**	**	**	ns	**	1.0
	01/21	1083	1124	**	1095	1116	1098	1104	ns	ns	ns	ns	ns	3.6
	01/44	3285	3305	ns	3280	3313	3291	3297	ns	ns	ns	ns	ns	3.4
FI	01/07	182	184	**	182	183	181	184	**	ns	ns	*	**	1.0
	01/14	544	545	ns	577	540	534	529	**	**	**	**	**	0.7
	01/21	1267	1277	ns	1328	1286	1241	1233	**	**	**	*	ns	4.4
	01/44	5735	5803	**	5825	5764	5753	5735	*	**	**	ns	ns	1.6
WG	01/07	188	198	**	190	194	194	194	**	**	**	ns	ns	1.2
	01/14	537	563	**	533	553	554	560	**	**	**	ns	**	0.52
	01/21	1040	1077	**	1050	1071	1053	1059	ns	ns	ns	ns	ns	3.5
	01/44	3241	3259	ns	3234	3268	3246	3252	ns	ns	ns	ns	ns	3.5
FCR	01/07	0.965	0.928	**	0.960	0.942	0.934	0.950	**	**	**	ns	**	1.5
	01/14	1.015	0.970	**	1.084	0.977	0.964	0.944	**	**	**	**	**	0.8
	01/21	1.219	1.187	*	1.265	1.202	1.181	1.165	*	**	**	ns	ns	4.7
	01/44	1.772	1.783	ns	1.804	1.764	1.774	1.766	ns	**	ns	ns	ns	4.1
M	01/44	7.14	4.94	**	6.29	6.92	5.94	5.00	ns	ns	ns	ns	ns	—

† Blood plasma inclusion in the diet: T1 – no plasma inclusion, T2 – 1% plasma until 7 d, T3 – 1% plasma until 7 d and 0.5% until 14 d, T4 – 1% plasma until 7 d, 0.5% until 14 s, and 0.25% until 21 d. ‡Effect of breeder age. §Effect of Inclusion of plasma in the diet. *P < 0.05, **P < 0.01, ns = not significant. ¶Orthogonal contrast: C1 - T1 vs (2-4), C2 – T1 vs T2, C3 – T2 vs T3, C4 – T3 vs T4.

The higher final body weight observed in broilers from older breeder hens can be attributed to their greater initial body weight at the start of the experiment. This difference is related to physiological changes that occur with advancing breeder age, such as increased intervals between ovulations and reduced laying rates, resulting in greater yolk deposition per egg (Zakaria et al., 1983). This phenomenon leads to increased egg size and weight, providing a larger amount of yolk and, consequently, higher chick body weight at hatch.

Chicks hatched from larger eggs or older breeders start with a higher body weight, but first-wk feed intake may be similar across breeder ages; nevertheless, differences in intestinal development and hatch weight help explain variations in early weight gain (Ipek and Sözcü, 2015). Moreover, these broilers possess a more developed gastrointestinal tract immediately after hatching, facilitating adaptation to feeding and contributing to improved performance during the first wk of life (Cardeal et al., 2021). This highlights the importance of initial chick weight at 1-d-old for subsequent broiler performance.

Maiorka et al. (2000) demonstrated that, within the first 24 h post-hatch and in the absence of feed, chicks from 60-wk-old breeders exhibited greater length and relative weight of the small intestine compared to those from younger breeders.

Mortality rates were higher in broilers from younger breeders. This may be related to lower transfer of maternal antibodies, since passive immunity via the yolk sac is crucial for early protection (Haems et al., 2024).

The inclusion of spray-dried plasma in the diet positively influenced final body weight and weight gain up to 14 d. Feed intake was significantly affected across all evaluated phases, as was feed conversion up to 21 d. Treatments containing plasma yielded the best results for final weight, weight gain, and feed conversion. Although feed intake was lower in these treatments, performance variables were not compromised, indicating greater efficiency in nutrient absorption and utilization.

Mortality rate was not affected by the feeding program. These findings suggest that plasma is a promising additive to enhance broiler performance, particularly during the early d of life, a critical period for infection susceptibility.

The superior performance may be related to the protein content and functional fractions of SDP, including immunoglobulins (predominantly IgG) and albumin, which support mucosal integrity and barrier function (Pierce et al., 2005; Campbell et al., 2010).

Under the conditions of this study, characterized by reused litter and high population density, the inclusion of plasma may have contributed to preventing pathogen adhesion to the intestinal mucosa, thereby improving zootechnical performance. According to Barbosa et al. (2007), plasma reduces antigen exposure and pro-inflammatory cytokine production, supporting performance (Campbell et al., 2008; 2010).

There is evidence that SDP modulates inflammatory responses and preserves the intestinal barrier, reducing pro-inflammatory cytokine responses and disease-related damage (Campbell et al., 2008; 2010). The contribution of functional proteins such as immunoglobulins and albumin to improved nutrient use is well documented (Pierce et al., 2005).

The results of this study are consistent with findings from Bhuiyan et al. (2014), who also reported improved performance and feed conversion in broilers fed plasma. The feeding program, that included 1% plasma until seven d and 0.5% until 14 d, yielded the best results, with significant effects observed up to 14 d.

Contrast analysis between treatments confirmed that plasma inclusion promoted lower feed intake, better feed conversion, greater weight gain, higher final body weight, and lower average mortality.

These findings underscore the need for growth-promoting additives, particularly in systems using reused litter. Spray-dried plasma emerges as a viable and effective alternative to antibiotic growth promoters in broiler diets.

A significant interaction (*P* < 0.05) between breeder age and dietary inclusion of spray-dried plasma was observed for final body weight and weight gain up to 14 d (Table 4). Feed intake was influenced throughout all phases of the rearing period. Notably, the highest mortality rate occurred in the treatment with 1.0% plasma inclusion up to seven d of age.

**Table 4:** Interaction (AxP) between breeder age (A) and plasma supplementation in the diet of broiler chickens (P) from 1 to 44 d of age for final body weight (FBW, g), feed intake (FI, g), weight gain (WG, g), conversion ratio (FCR), and mortality (M, %) of broiler chickens from 1 to 44 d fed diets containing blood plasma.

Parameters	Breeder age – 36 wk					†Breeder age – 56 wk				^‡^AxP
	†Inclusion of plasma in the diet					†Inclusion of plasma in the diet				
	D	T1	T2	T3	T4	T5	T6	T7	T8	
FBW	01/07	226^c^	234^b^	232^b^	234^b^	244^a^	244^a^	246^a^	244^a^	**
	01/14	559^g^	587^f^	586^f^	589^e^	597^d^	609^c^	612^b^	621^a^	**
	01/21	1084	1086	1080	1082	1107	1146	1117	1125	ns
	01/44	3267	3290	3290	3292	3293	3336	3292	3303	ns
FI	01/07	182	185	176	184	182	181	187	185	**
	01/14	578	545	537	518	576	535	532	539	**
	01/21	1329	1269	1036	1231	1328	1303	1243	1235	**
	01/44	5806	5699	5731	5706	5844	5828	5776	5764	**
WG	01/07	183	191	189	190	197	198	199	198	**
	01/14	515	544	543	546	550	562	566	574	**
	01/21	1040	1043	1036	1039	1061	1099	1070	1078	ns
	01/44	3223	3247	3247	3248	3246	3289	3245	3245	ns
FCR	01/07	0.990	0.960	0.930	0.966	0.924	0.916	0.940	0.934	ns
	01/14	1.121	1.003	0.989	0.948	1.048	0.951	0.940	0.939	ns
	01/21	1.278	1.217	1.197	1.185	1.252	1.118	1.165	1.145	ns
	01/44	1.805	1.756	1.766	1.759	1.803	1.773	1.782	1.772	ns
M	01/44	6.66	9.26	7.07	5.55	5.92	4.58	4.81	4.45	**

† Blood plasma inclusion in the diet: T1 – no plasma inclusion, T2 – 1% plasma until 7 d, T3 – 1% plasma until 7 d and 0.5% until 14 d, T4 – 1% plasma until 7 d, 0.5% until 14 d, and 0.25% until 21 d. ‡Interaction between breeder age and plasma inclusion in the diet. *P < 0.05, **P < 0.01, ns = not significant. a,b Tukey test.

Plasma supplementation in the diets of chicks from younger breeders (36 wk) resulted in improved final body weight, particularly during the initial phase. From 14 d onward, increased body weight was observed in broilers from both 36- and 56-wk-old breeders. Feed intake was highest in broilers from 36-wk-old breeders that did not receive plasma, while those supplemented with plasma regardless of breeder age exhibited enhanced weight gain.

These findings suggest that, independent of breeder age, plasma inclusion positively influenced broiler performance up to 14 d, serving as a valuable nutritional strategy, especially for chicks from younger breeders. Although the mechanisms by which plasma exerts these effects remain partially understood, its composition containing approximately 22.5% immunoglobulins and 28.0% albumin (Pierce et al., 2005) is believed to play a central role.

Under the conditions of this study, which included reused litter and high stocking density, broilers were exposed to elevated levels of excreta and microbial contamination, contributing to environmental stress and increased susceptibility to disease. Nevertheless, plasma supplementation improved final body weight even in broilers from younger breeders housed under these challenging conditions, supporting its role in maintaining early growth and development.

This rationale aligns with previous research indicating that the benefits of plasma supplementation particularly in terms of weight gain and feed intake are more pronounced under sanitary challenge than in clean environments (Campbell et al., 2010).

The results of this study are consistent with findings from Barbosa et al. (2013), who also reported significant improvements in broiler performance following dietary inclusion of plasma.

According to the histological data presented in Table 5, both breeder age and dietary inclusion of spray-dried plasma significantly influenced (*P* < 0.05) villus height, crypt depth, villus-to-crypt ratio, and the number of goblet cells.

**Table 5:** Intestinal parameters (Villi, µm, Crypt, µm, Villus:crypt ratio, and Goblet cells, und.) of broiler chickens from 1 to 44 d with dietary inclusion of blood plasma.

Parameters	D	Breeder age			†Inclusion of plasma in the diet					^¶^Contrasts				CV
		wk		*^‡^P*										
		36	56		T1	T2	T3	T4	*^§^P*	C1	C2	C3	C4	
Villi	01/07	1383	1487	**	1387	1405	1537	1426	**	**	**	**	**	0.21
	01/14	1883	1925	**	1893	1839	2033	1851	**	**	**	**	**	0.12
	01/21	1923	1965	**	1972	1881	2022	1899	**	**	**	**	**	0.11
	01/44	1966	2006	**	2050	1928	2013	1950	**	**	**	**	**	0.10
Crypt	01/07	231	240	**	235	215	247	242	**	ns	**	**	**	0.56
	01/14	238	239	ns	248	231	246	230	**	**	**	**	**	0.63
	01/21	255	243	**	265	238	247	247	**	**	**	**	ns	0.96
	01/44	269	246	**	277	243	244	265	**	**	**	*	**	0.73
*Villus: crypt*	01/07	6.03	6.21	**	5.90	6.52	6.21	5.89	**	**	**	**	**	0.55
	01/14	7.89	8.04	**	7.62	7.96	8.24	8.03	**	**	**	**	**	0.64
	01/21	7.54	8.07	**	7.45	7.90	8.17	7.69	**	**	**	**	**	0.98
	01/44	7.35	8.15	**	7.53	7.93	8.23	7.34	**	**	**	**	**	0.76
Goblet cells	01/07	52.01	47.46	**	41.14	56.99	55.14	45.75	**	**	**	**	**	15.3
	01/14	88.87	82.87	**	80.17	81.39	92.89	89.03	**	ns	ns	ns	ns	12.7
	01/21	70.76	65.44	**	61.00	69.35	74.35	67.71	**	ns	ns	ns	ns	9.2
	01/44	52.59	50.60	ns	54.96	49.75	50.35	51.32	ns	ns	ns	ns	ns	18.5

† Blood plasma inclusion in the diet: T1 – no plasma inclusion, T2 – 1% plasma until 7 d, T3 – 1% plasma until 7 d and 0.5% until 14 d, T4 – 1% plasma until 7 d, 0.5% until 14 d, and 0.25% until 21 d. ‡Effect of breeder age. §Effect of Inclusion of plasma in the diet. *P < 0.05, **P < 0.01, ns = not significant. ¶Orthogonal contrast: C1 - T1 vs (2-4), C2 – T1 vs T2, C3 – T2 vs T3, C4 – T3 vs T4.

Chicks from older breeders exhibited greater villus height compared to those from younger breeders across all evaluated stages. Crypt depth was also affected by breeder age between 7 and 21 d, with broilers from older breeders showing shallower crypts during this period.

Broiler chickens from older breeder hens showed a better villus-to-crypt ratio across all evaluation phases. The highest number of goblet cells was observed in broilers from 36-wk-old breeders, with these results evident up to 21 d. broilers from older breeders tend to have a more developed gastrointestinal tract post-hatch, as demonstrated by Maiorka et al. (2004), which may explain the greater villus height, shallower crypt depth, and improved villus-to-crypt ratio in these broilers.

Additionally, broilers from younger breeders receive fewer maternal antibodies, which may facilitate bacterial adhesion to the intestinal wall. Given the high sanitary challenge in this experiment, this could explain the poorer histological outcomes observed in these broilers.

The presence of bacteria on the mucosa leads to damage to the intestinal villi. As a result, crypt cells differentiate and migrate toward the villi to repair the damage, causing deeper crypts (Maiorka et al., 2003).

Crypts in broilers from younger breeders were deeper during the last two rearing phases. According to Uni et al. (1998), increased crypt depth reflects a higher rate of epithelial cell proliferation, a physiological response to maintain villus structure and preserve absorptive surface area.

It is worth noting that in later rearing phases, the litter contained more excreta, creating a favorable environment for bacterial proliferation. Consequently, broilers faced greater sanitary challenges. Due to lower maternal antibody transfer, chicks from younger breeders required a higher number of immune cells.

Shallower crypts indicate less morphological damage to the intestinal wall. Moreover, reduced crypt depth implies lower energy, and protein demands for tissue renewal, thereby improving broilers efficiency. An optimal villus-to-crypt ratio is achieved when villi are tall, and crypts are shallow. A higher ratio enhances nutrient absorption and reduces energy losses from cellular turnover (Arruda et al., 2008), a pattern more evident in broilers from older breeders.

Regarding goblet cell count, a higher number was found in broilers from younger breeders, suggesting immune system activation to maintain absorptive area. These broilers also showed deeper crypts, indicating greater impact from sanitary challenges compared to those from older breeders.

These findings align with Fernandes et al. (2014), who studied the influence of breeder age on organ biometrics and intestinal mucosa morphology in newly hatched chicks. Using eggs from breeders aged 32, 40, 48, and 56 wk, they also observed deeper crypts in chicks from younger breeders.

The inclusion of blood plasma in the diet significantly influenced (*P* < 0.05) all evaluated parameters across all phases, except for goblet cell count (*P* > 0.05), which was only affected up to 21 d. Treatments containing plasma yielded the most favorable histological results.

Plasma-supplemented diets proved more efficient. Even when villus height was lower than in the control group, crypt depth was reduced, and the villus-to-crypt ratio was consistently higher. Goblet cell counts also improved, indicating reduced intestinal wall aggression.

The positive effects of plasma supplementation may be linked to its lower stimulation of the immune system, due to the presence of immunoglobulins, especially IgG, a key antibody in immune responses. This enhances local immunity and reduces bacterial proliferation on the intestinal mucosa, preventing villus damage (Barbosa et al., 2013).

The control group (without plasma) showed deeper crypts, particularly in the final evaluation phase, likely due to increased sanitary stress from higher excreta accumulation, bacterial load, and reduced space caused by increased body weight.

These results support findings from other authors regarding intestinal wall integrity and plasma supplementation (Barbosa et al., 2012).

The treatment with plasma supplementation up to 14 d (1% and 0.5%, respectively) showed the best intestinal parameter outcomes, confirmed through dietary program contrasts.

There was a significant interaction (*P* < 0.05) between breeder age and plasma inclusion for intestinal parameters (Table 5), including villus height, crypt depth, and villus-to-crypt ratio from 14 to 44 d. Goblet cell count was influenced by breeder age across all phases.

Plasma supplementation improved intestinal parameters and helped maintain barrier integrity in broilers from both 36- and 56-wk-old breeders. This is evident in the villus-to-crypt ratio, which was consistently better in plasma-treated groups.

Goblet cell counts were higher up to 21 d of age in all plasma-treated groups. Since these treatments also showed improved villus-to-crypt ratios, it can be inferred that plasma inclusion enhances immune cell numbers without triggering full immune activation.

At 44 d, the phase with the highest sanitary challenge growth, non-SDP treatments showed deeper crypts and elevated goblet cell counts, indicating a need for immune cell production and crypt-to-villus migration to repair damage.

According to Campbell et al. (2008), stress and antigen exposure activate the immune system and stimulate pro-inflammatory cytokines in the brain, which interact with insulin-like growth factors to suppress cell growth. Recent evidence suggests that plasma reduces excessive cytokine responses, mitigating disease-related damage.

These findings are crucial in demonstrating that even broilers from younger breeders can improve digestive capacity with plasma supplementation, enhancing performance parameters and supporting commercial production.

Breeder age significantly influenced (*P* < 0.05) eviscerated carcass weight, breast weight, and breast yield (Table 6). Broilers from 56-wk-old breeders had higher carcass weights, possibly due to starting the experiment with greater body mass, increased early feed intake, and/or better feed conversion.

**Table 6:** Carcass parameters of broiler chickens from 1 to 44 d fed diets containing spray-dried porcine plasma (SDP). Fasted body weight (FBW, kg), Eviscerated carcass weight (ECW, kg), Eviscerated carcass yield (ECY, %), Breast weight (BW, kg), Breast yield (BY, %), Thigh and drumstick weight (TDW, kg), Thigh and drumstick yield (TDY, %), Abdominal fat weight (AFW, kg) Abdominal fat yield (AFY, %).

Parameters	Breeder age			†Inclusion of plasma in the diet					¶Contrasts				CV
	wk		^‡^*P*										
	36	56		T1	T2	T3	T4	*^§^P*	C1	C2	C3	C4	
FBW	3.284	3.306	ns	3.279	3.310	3.289	3.303	ns	ns	ns	ns	ns	3.4
ECW	2.518	2.566	**	2.519	2.536	2.573	2.537	*	*	ns	ns	**	2.2
ECY	76.70	77.68	ns	76.90	76.64	78.32	76.86	ns	ns	ns	**	**	3.40
BW	0.898	0.873	**	0.892	0.903	0.865	0.886	**	ns	ns	**	*	3.0
BY	27.37	26.42	**	27.20	27.28	26.33	26.83	*	**	ns	*	ns	3.6
TDW	0.644	0.651	ns	0.648	0.651	0.642	0.648	ns	ns	ns	ns	ns	3.2
TDY	19.62	19.69	ns	19.77	19.68	19.54	19.63	ns	ns	ns	ns	ns	3.6
AFW	63.79	64.26	ns	63.82	63.57	64.26	64.39	ns	ns	ns	ns	ns	3.1
AFY	1.944	1.946	ns	1.947	1.922	1.956	1.953	ns	ns	ns	ns	ns	4.7

† Blood plasma inclusion in the diet: T1 – no plasma inclusion, T2 – 1% plasma until 7 d, T3 – 1% plasma until 7 d and 0.5% until 14 d, T4 – 1% plasma until 7 d, 0.5% until 14 d, and 0.25% until 21 d. ‡Effect of breeder age. §Effect of Inclusion of plasma in the diet. *P < 0.05, **P < 0.01, ns = not significant.

¶ Orthogonal contrast: C1 - T1 vs (2-4), C2 – T1 vs T2, C3 – T2 vs T3, C4 – T3 vs T4.

However, the best results for breast weight and yield were observed in broilers from 36-wk-old breeders. Lower breast yield in other groups was offset by greater meat deposition in other cuts, such as thighs and drumsticks, resulting in higher yields for these parts. This may be linked to genetic factors favoring breast meat deposition in broilers from younger breeders.

The inclusion of blood plasma in the diet significantly influenced (*P* < 0.05) eviscerated carcass weight, breast weight, and breast yield. Carcasses from broilers fed plasma-containing diets were heavier, likely due to improved nutrient utilization, as evidenced by both performance and histological data. The highest breast weight and yield were observed in broilers that received plasma supplementation up to 14 d.

Additionally, this treatment also resulted in greater thigh and drumstick weights, as shown by the contrast between treatments 2 and 3 namely, 1.0% plasma until 7 d of age versus 1.0% plasma until 7 d followed by 0.5% plasma until 14 d.

Similar results for cut yields under different stocking densities were reported by Moreira et al. (2004). These authors concluded that offspring from younger breeders showed higher breast weight and breast yield compared to those from older breeders.

The treatment that showed the best results was the one containing 1.0% blood plasma in the diet until seven d of age, as verified through contrast analysis between treatments. In addition to presenting higher breast weight and yield, it also showed greater thigh and drumstick weight and yield, along with lower abdominal fat values.

Bregendahl et al. (2005) observed an increase in breast weight and yield in animals that received plasma in their diet. According to the authors, the effect of plasma on feed intake may have manifested as early as the third d of age, which could explain the improvement in breast yield.

There was an interaction between breeder age and blood plasma inclusion in the diet on carcass parameters (Table 7), specifically for breast weight and yield.

**Table 7:** Carcass Parameters of Broiler Chickens from 1 to 44 d: Interaction Between Breeder Age and Blood Plasma Inclusion in the Diet. Fasted body weight (FBW, kg), Eviscerated carcass weight (ECW, kg), Eviscerated carcass yield (ECY, %), Breast weight (BW, kg), Breast yield (BY, %), Thigh and drumstick weight (TDW, kg), Thigh and drumstick yield (TDY, %), Abdominal fat weight (AFW, kg) Abdominal fat yield (AFY, %).

Parameters	Breeder age – 36 wk				†Breeder age – 56 wk				^‡^AxP
	†Inclusion of plasma in the diet				†Inclusion of plasma in the diet				
	T1	T2	T3	T4	T5	T6	T7	T8	
FBW	3.266	3.290	3.289	3.292	3.293	3.339	3.288	3.315	**
ECW	2.503^b^	2.510^b^	2.527^b^	2.531^b^	2.534^b^	2.574^ab^	2.620^a^	2.543^ab^	**
ECY	76.73	76.29	76.83	76.97	77.05	77.15	79.80	76.75	ns
BW	0.914^a^	0.921^a^	0.873^b^	0.884^ab^	0.871^b^	0.877^ab^	0.857^b^	0.887^ab^	ns
BY	28.04^a^	27.99^a^	26.55^b^	26.89^ab^	26.46^b^	26.27^b^	26.10^b^	26.77^b^	**
TDW	0.643	0.645	0.641	0.645	0.652	0.659	0.643	0.650	**
TDY	19.71	19.62	19.51	19.63	19.82	19.76	19.58	19.62	**
AFW	63.18	62.80	64.79	64.45	64.39	64.68	63.73	64.34	**
AFY	1.94	1.91	1.97	1.96	1.96	1.94	1.94	1.94	**

† Blood plasma inclusion in the diet: T1 – no plasma inclusion, T2 – 1% plasma until 7 d, T3 – 1% plasma until 7 d and 0.5% until 14 d, T4 – 1% plasma until 7 d, 0.5% until 14 d, and 0.25% until 21 d. ‡Interaction between breeder age and plasma inclusion in the diet. *P < 0.05, **P < 0.01, ns = not significant. a,b Tukey test.

Broilers from 36-wk-old breeder hens that received plasma supplementation in their diet until seven d of age showed heavier breast weights. In this treatment, higher weights were also observed for thighs and drumsticks, along with lower abdominal fat weight. As for breast yield, the highest values were found in treatments consisting of broilers from 36-wk-old breeders that did not receive spray-dried porcine plasma (SDP) in their diet.

It is worth noting that the inclusion of plasma in the diet during the first d of life resulted in greater weight gain during the phase. However, increased breast meat deposition should not be attributed solely to plasma inclusion, as although the treatment with plasma until seven d of age showed a higher value compared to the treatment without plasma, no statistical difference was observed.

Furthermore, based on data regarding the influence of breeder age on carcass parameters (Table 7), it was observed that broilers from younger breeders deposit more breast meat than broilers from older breeders, regardless of blood plasma inclusion in the diet.

In the case of broilers from 56-wk-old breeders, an increase in breast weight was observed with plasma supplementation, with treatments receiving plasma until seven d and those receiving it until 21 d showing heavier breasts.

These results are consistent with reports of increased breast yield in broilers fed SDP (Bregendahl et al., 2005).

No isolation of Escherichia coli or Salmonella spp. (Table 8) was obtained on the first d of life in any of the biological materials studied, regardless of breeder age or blood plasma supplementation in the diet.

**Table 8:** Isolation of Escherichia coli and Salmonella spp. in Broiler Chickens from 1 to 44 d with Blood Plasma Inclusion in the Diet.

Isolation at d 1		Breeder age – 36 wk				Breeder age – 56 wk			
		†Inclusion of plasma in the diet							
Isolation	Biological Material	T1	T2	T3	T4	T5	T6	T7	T8
*Escherichia coli*	Poultry litter	-	-	-	-	-	-	-	-
	Cloacal Swab	-	-	-	-	-	-	-	-
	Cecal Content	-	-	-	-	-	-	-	
*Salmonella* spp*.*	Poultry litter	-	-	-	-	-	-	-	-
	Cloacal Swab	-	-	-	-	-	-	-	-
	Cecal Content	-	-	-	-	-	-	-	-
	Isolation at d 14	T1	T2	T3	T4	T5	T6	T7	T8
*Escherichia coli*	Poultry litter	-	-	-	-	-	-	-	-
	Cloacal Swab	-	*	-	-	-	-	-	*
	Cecal Content	-	*	*	*	-	-	*	*
*Salmonella* spp*.*	Poultry litter	-	-	-	-	-	-	-	-
	Cloacal Swab	-	-	-	-	-	-	-	-
	Cecal Content	-	-	-	-	-	-	-	*
	Isolation at d 14	T1	T2	T3	T4	T5	T6	T7	T8
*Escherichia coli*	Poultry litter	*	-	-	-	-	-	-	-
	Cloacal Swab	-	-	-	-	-	-	-	-
	Cecal Content	-	-	-	-	-	-	-	-
*Salmonella* spp*.*	Poultry litter	-	-	*	-	*	-	-	-
	Cloacal Swab	-	-	-	-	-	-	-	-
	Cecal Content	-	-	-	-	-	*	-	-
	Isolation at d 21	T1	T2	T3	T4	T5	T6	T7	T8
*Escherichia coli*	Poultry litter	-	-	-	-	*	*	-	-
	Cloacal Swab	*	-	-	-	-	-	-	-
	Cecal Content	-	-	-	-	-	-	-	-
*Salmonella* spp*.*	Poultry litter	-	-	-	*	*	*	-	*
	Cloacal Swab	*	*	-	-	-	-	-	*
	Cecal Content	-	-	-	-	-	-	-	*
	Isolation at d 44	T1	T2	T3	T4	T5	T6	T7	T8
*Escherichia coli*	Poultry litter	-	-	-	-	-	-	-	-
	Cloacal Swab	*	-	-	*	-	*	*	-
	Cecal Content	-	*	-	*	-	-	-	*
*Salmonella* spp*.*	Poultry litter	*	-	-	*	*	*	*	-
	Cloacal Swab	-	*	*	-	*	-	-	*
	Cecal Content	*	-	*	-	*	*	*	*

† Blood plasma inclusion in the diet: T1 – no plasma inclusion, T2 – 1% plasma until 7 d, T3 – 1% plasma until 7 d and 0.5% until 14 d, T4 – 1% plasma until 7 d, 0.5% until 14 d, and 0.25% until 21 d. (-) Absence of Escherichia coli and Salmonella spp. (*) Presence of Escherichia coli and Salmonella spp.

At seven d of age, Escherichia coli was isolated in broilers both young and old breeders, in both cloacal swabs and cecal contents. For Salmonella spp., presence was only detected in cecal contents of chicks from older breeders that received plasma supplementation in the diet. This can be explained by the fact that *Escherichia coli* is a normal inhabitant of the gastrointestinal tract (Carter, 1988).

At 14 d, Escherichia coli was found in the litter of broilers from 36-wk-old breeders that did not receive plasma in the diet, and Salmonella spp. was detected in the treatment consisting of broilers from 36-wk-old breeders that received 1.0% plasma until seven d, followed by 0.5% until 14 d.

In treatments involving 56-wk-old breeders, Salmonella spp. was observed both in the litter and in cecal contents, in the treatment without plasma supplementation as well as in the treatment that received 1.0% blood plasma until seven d.

At 21 d, Escherichia coli was isolated from cloacal swabs of broilers from 36-wk-old breeder hens that did not receive blood plasma in their diet, and from the litter in treatments consisting of broilers from 56-wk-old breeders without plasma supplementation and those that received 1.0% plasma until seven d.

As for Salmonella spp., it was detected in cloacal swabs of broilers from 36-wk-old breeders without plasma supplementation and those that received 1.0% plasma until seven d, as well as in treatments with broilers from 56-wk-old breeders that received plasma until 21 d, in all biological materials. Salmonella spp. was also found in the litter of treatments with broilers from 36-wk-old breeders that received plasma until 21 d, and in treatments with broilers from 56-wk-old breeders that did not receive plasma and those that received plasma until seven d.

At 44 d, *Escherichia coli* was detected in cloacal swabs of broilers from 36-wk-old breeders without plasma supplementation and those that received plasma until 21 d, as well as in treatments with broilers from 56-wk-old breeders that received plasma until seven and 14 d, respectively. In cecal contents, Escherichia coli was found in treatments with broilers from 36-wk-old breeders that received plasma until seven and 21 d, and in broilers from 56-wk-old breeders that received plasma until 21 d.

The presence of *Salmonella spp*. was verified in all biological materials at 44 d, being found in the litter of treatments with broilers from 36-wk-old breeders that did not receive plasma at any stage and those that received plasma until 21 d. In treatments with broilers from 56-wk-old breeders, *Salmonella spp*. was present in groups that did not receive plasma, those that received it until seven d, and those that received plasma until 14 d.

In cloacal swabs, Salmonella spp. was detected in treatments with broilers from 36-wk-old breeders that received plasma until seven and 14 d, respectively, and in treatments with broilers from 56-wk-old breeders that did not receive plasma and those that received it until seven and 14 d.

In cecal contents, Salmonella spp. was observed in treatments with broilers from 36-wk-old breeders without plasma inclusion and those that received plasma until 14 d, and in all treatments with broilers from 56-wk-old breeders.

It is important to highlight that in this study, the animals were challenged by the use of recycled litter and a higher number of broilers both inside the pens and loose within the barn, which contributed to the increased presence of bacteria.

However, based on the results obtained for performance and histological variables, the presence of bacteria in treatments with plasma supplementation, regardless of breeder age, did not affect intestinal parameters, resulting in better performance of these broilers.

According to Pierce et al. (2005), the benefits associated with plasma inclusion regarding bacterial adhesion to the intestinal epithelium are attributed to the activity of glycoproteins and immunoglobulins present in plasma, which reinforce epithelial protection against Escherichia coli.

Furthermore, plasma improves performance by enhancing the animal’s immunocompetence through the immunoglobulins it contains and reduces immune system exposure to antigens, leading to lower production of pro-inflammatory cytokines (Campbell et al., 2008, 2010). Studies involving pathogenic bacteria such as Escherichia coli and Salmonella spp. have shown reduced mortality and morbidity when animal plasma was added to swine diets (Bailey et al., 2025). According to Campbell et al. (2008), the effects of plasma are more evident when animals are subjected to high pathogen exposure conditions.

## Conclusion

The inclusion of spray-dried porcine plasma (SDP) in diets of broiler, originating from breeder hens of different ages and raised on recycled litter, improves the broilers overall performance. It is recommended to use spray-dried porcine plasma (SDP) up to seven d for broilers from older breeder hens, and up to 21 d for broilers from younger breeder hens.

## CRediT authorship contribution statement

**Luzia Trajano da Silva:** Conceptualization, Formal analysis, Investigation, Supervision, Writing – original draft, Writing – review & editing, Data curation. **Adiel Vieira de Lima:** Conceptualization, Formal analysis, Writing – review & editing, Data curation. **Danilo Vargas Gonçalves Vieira:** Conceptualization, Data curation, Formal analysis, Writing – review & editing. **Danilo Teixeira Cavalcante:** Conceptualization, Formal analysis, Writing – review & editing. **Matheus Ramalho de Lima:** Conceptualization, Formal analysis. **Apolônio Gomes Ribeiro:** Conceptualization, Data curation, Formal analysis, Writing – review & editing. **Carlos Henrique do Nascimento:** Conceptualization, Formal analysis. **Paloma Eduarda Lopes de Souza:** Conceptualization, Formal analysis. **Aline Beatriz Rodrigues:** Conceptualization, Formal analysis. **Ricardo Romão Guerra:** Conceptualization, Formal analysis, Writing – review & editing. **Leonardo Augusto Fonseca Pascoal:** Conceptualization, Formal analysis, Writing – review & editing. **Lucas Rannier Ribeiro Antonino Carvalho:** Conceptualization, Formal analysis, Writing – review & editing. **Fernando Guilherme Perazzo Costa:** Conceptualization, Data curation, Funding acquisition, Methodology, Project administration, Validation, Writing – original draft, Writing – review & editing.

## Disclosures

The authors declare that they have no known competing financial interests or personal relationships that could have appeared to influence the work reported in this paper.
